# Diagnosis and Surgical correction of salivary affections in buffaloes (Bubalus bubalis); a retrospective study

**DOI:** 10.1186/s12917-023-03755-5

**Published:** 2023-10-19

**Authors:** Esam Mosbah, Marwa Abass, Khaled Abouelnasr, Mohamed Salem

**Affiliations:** Department of Surgery, Anesthesiology, and Radiology, Faculty of Veterinary Medicine, Mansoura University, Mansoura, 35516 Egypt

**Keywords:** Buffaloes, Salivary fistula, Ectasia, Mucocele, Cervical sialocele

## Abstract

**Aim:**

This study aimed to describe the diagnosis and treatment of various surgical salivary affections in buffaloes.

**Materials and methods:**

This study included 135 buffaloes examined at Dakahlia Governorate between 2011 and 2022 suffering from various surgical salivary affections. The recorded surgical affections had salivary fistula (n = 44), ectasia of Stenson’s duct (n = 11), ranula/mucocele (n = 46), and cervical sialocele (n = 34). The buffaloes were sedated using an intramuscular injection of xylazine (0.05 mg/kg) and local infiltration analgesia of lidocaine for specific surgical interventions.

**Results:**

The salivary duct fistula cases were surgically corrected using a retrograde infusion of povidone-iodine into the duct and its double ligation with Prolene following fistulectomy. Intraoral marsupialization was done in buffaloes suffering from ectasia of the parotid duct. The mucocele /ranula was surgically incised with daily flushing with povidone-iodine. The cervical sialocele was treated by giving an elliptical excision on the sialocele, and sialoadenectomy of the mandibular salivary gland was performed to facilitate dynamic fluid/saliva drainage. A 92.5% of diseased buffaloes showed an uneventful recovery without any postoperative complications after the first treatment, whereas 7.5% of animals tended to recur. The most common and almost equally distributed salivary affections recorded in adult buffaloes were parotid duct fistula, mucocele, and cervical sialocele. The Stenson’s duct ectasia was commonly registered in calves, being congenital.

**Conclusion:**

Ranula was the most common salivary affection encountered in adult buffaloes, closely followed by parotid duct fistulae and cervical sialoceles. Stenson’s duct ectasia was the least encountered salivary affection in calves and was congenital. All salivary affections were corrected easily and safely, with satisfactory outcomes.

## Background

The salivary glands are multifunctional paired structures found in domestic animals. There are three pairs of salivary glands: the submandibular, sublingual, and parotid glands. The submandibular gland is the largest, while the sublingual is the smallest [1, 2]. The parotid salivary gland is situated under the ear relative to the caudal edge of the mandible and drains through the Stenson’s duct, which passes ventrally and cranially on the deep face of the caudal part of the mandible, crosses the cheek superficially just cranial to the masseter muscle and ultimately penetrates the cheek near the upper third or fourth cheek tooth [1, 2]. The submandibular salivary gland is situated under the parotid gland, just caudal to the mandible. Its duct, called the Wharton duct, passes forward and medial to the mandible and runs on the floor of the mouth cavity to open ventrally to the tongue in close association with the sublingual duct at the caruncula sublingualis slightly anterolateral to the frenulum linguae [2].

Salivary glands play an essential role in the oral cavity by producing saliva. However, various diseases, such as tumors, infections, physical traumas, fistulas, ectasia, sialoliths, ranulas, and sialoceles, can alter the functionality of these glands, significantly impacting the quality of life of buffaloes [1]. In domestic animals, numerous salivary gland affections have been reported in the literature, including sialoliths in camels and donkeys [3], salivary fistulas in bullocks [4], sialoceles in buffaloes [5], ectasia of the parotid duct in buffaloes [6], and ranulas in buffaloes [7]. These affections can be caused by penetrating injuries, facial trauma, infections, tumors, or congenital abnormalities, leading to difficulty feeding [8]. Sialectasis is defined as an abnormal expansion of Stenson’s duct, reported in both human and animal fields [9, 10]. Salivary fistulas can occur due to obstruction of the salivary gland duct by foreign objects, such as rumen cud, grass spines, blood clots, or stones, leading to its distension with saliva and eventual rupture forming a salivary cyst [4, 11]. Ranulas are accumulations of extra-glandular saliva at the floor of the mouth, blocking the salivary gland and forming a retention transparent cyst under the tongue that interferes with standard feeding [12]. Ranulas typically develop following intraoral trauma caused by ingesting rough grass particles, high pressure, blunt trauma to the salivary duct, or sialoliths [6, 13]. Cervical sialoceles result from the rupture of the salivary gland or its duct, accumulating saliva in the surrounding tissue, surrounded by granulation tissue [14]. Thus, the present retrospective study was to account for salivary tract affections, their diagnosis, and treatment in Egyptian buffaloes (Bubalus bubalis).

## Results

### Animal and physical examination

Four salivary affections were registered in 135 buffaloes, 26 calves (15 males and 11 females), and 109 adult female buffaloes. Their weight, age, and occurrence % of the affection are described in (Table 1).

**Table 1:** Wight, age of buffaloes, and the percentage of occurrence of salivary affections

Affections	Age	Weight (kg)	Number (n)	Occurrence %
Salivary fistulae N = 44	4–24 months 4–8 years	90–175 350–830	6 calves (4 males and 2 females) 38 female buffaloes	4.4 28.2
Stenson’s duct ectasia/ Sialectasis N = 11	3–12 months 4–5 years	90–175 350–530	7 calves (3 males and 4 females) 4 female buffaloes	5.2 2.9
Ranula/ Sublingual mucocele N = 46	6–12 months 4–8 years	130–175 300–830	9 calves (4 males and 5 females) 37 female buffaloes	6.7 27.4
Cervical sialocele N = 34	9–18 months 3–9 years	165–205 400–930	4 male buffalo calves 30 female buffaloes	3 22.2

The physical parameters, such as the heart rate, respiratory rate, body temperature, and ruminal contraction, were within the normal range for all the buffaloes. In contrast, the skin fold test showed some degree of dehydration (7 ± 3 s).

All the diseased buffaloes had a fluid-filled structure or discharge, which increased during feeding and mastication. This fluid was honey-colored, seromucous, and dense in consistency. The laboratory analysis of this discharge revealed the presence of sodium carbonate (147.71 ± 0.69 mmol/dl) as the primary electrolyte and other traces of potassium (19.18 ± 0.44 mmol/dl), calcium (9.25 ± 0.09 mg/dl), magnesium (3.82 ± 0.2 mg/dl), and inorganic phosphates (10.54 ± 0.25 mg/dl). The collected fluid had an alkaline pH between 8 and 8.8.

### Parotid duct fistula

The parotid duct fistula was reported in forty-four buffaloes. This included six buffalo calves (4 males and 2 females) out of a total of 26 buffalo calves, with an occurrence percentage of 4.4%. The calves were aged between 4 and 24 months. Additionally, out of a total of 109 buffaloes examined, thirty-eight female buffaloes had an occurrence percentage of 28.2%. These female buffaloes were aged between 4 and 8 years. Twenty-one buffaloes (2 male buffalo calves, one female buffalo calve, and 18 female buffaloes) had a history of abscess drainage in the region two weeks prior. In contrast, the others had a history of direct trauma to the cheek. Salivary fistula appeared as a non-inflammatory swelling ranging from lemon to orange size at the cheek region with a ropy discharge, as noticed by the owners. All cases were chronic and associated with a small or narrow opening surrounded by hard tissue. The synovial discharge stopped completely after surgical intervention, and the wounds healed without complications. No cases showed a tendency to recur post-fistulectomy.

### Parotid duct ectasia/ sialectasis

Ectasia of the salivary duct was recorded in eleven cases: seven buffalo calves (three males and four females) aged 3–12 months, and four female buffaloes , aged 4–5 years. The occurrence rate of parotid duct ectasia was 5.2 % in buffalo calves and 2.9% in adult female buffaloes. Variable-sized swellings (ranging from 4 cm × 10 to 13 cm × 22 cm) appeared on the lateral aspect of the cheek. After needle aspiration of the contents, the swellings would be refilled within 15 min.

Recovery and gradual shrinkage of the swelling at the cheek were achieved within up to 20 days in all cases except for one male calf. The outcome percentage of buffalos’ recovery was 91% versus 9% recurrence (Table 2). In this calf, the swelling was still evident one month post-operation, so a 3 cm vertical incision was repeated for the second time, with a successful outcome.

**Table 2:** The outcomes percentage of salivary affection post-surgical treatment

Affections	Recovery% after first treatment	Recurrency occurrence %
Salivary fistulae N = 44	100% 6 calves (4 males and 2 females) 38 female buffaloes	0% No recurrence recorded
Stenson’s duct ectasia/ Sialectasis N = 11	91% 7 calves (3 males and 4 females) 3 female buffaloes	9% One male calf (re-marsupialization of swelling)
Ranula/ Sublingual mucocele N = 46	100% 9 calves (4 males and 5 females) 37 female buffaloes	0% No recurrency recorded
Cervical sialocele N = 34	73.5% 4 male buffalo calves 21 female buffaloes	26.5% Nine Female buffaloes (Sialadenectomy of mandibular salivary gland)

### Ranula/ mucocele

Ranula or mucocele was registered in nine buffalo calves (four males and five females) aged 6–12 months, with an occurrence percentage of 27.4%, and in thirty-seven adult female buffaloes aged 4–8 years old, with an occurrence percentage of 27.4%.

The affected buffaloes presented with ptyalism protruded tongues and slightly open mouths, while their physiological parameters remained normal. No vesicular lesions were observed inside the mouth cavity under the tongue close to the phrenum linguae. The clear, colorless fluid was aspirated and revealed synovia after laboratory analysis. Following marsupialization and destruction of the mucosa, no recurrence cases were recorded, and the outcome was satisfactory.

### Cervical sialoceles

Cervical sialoceles were observed in four male buffalo calves out of 26 buffalo calves, aged 9 -18 months and in thirty female buffaloes out of the 109 cases examined, aged 3–9 years.  The incidence rate for calves was 3% , while for female buffaloes was 22.2%. The condition presented as an irregular swelling of variable size located at the mandibular space with a gradual onset.

Exploratory percutaneous centesis revealed yellowish-colored fluid, which laboratory analysis confirmed to be saliva. The recovery percentage post incision and evacuation of swelling and a Penrose draining was 73.5%: four male buffalo calves and twenty-one adult female buffaloes (Table 2). The other nine female buffaloes showed a tendency for recurrence, with a percentage of 26.5%, so sialadenectomy (mandibular salivary gland excision) was performed.

## Discussion

Saliva plays a crucial role in maintaining the health of ruminants, facilitating mastication and deglutition, and helping to restore normal ruminal pH. The saliva contains essential electrolytes such as sodium, potassium, calcium, magnesium, and inorganic phosphorus. Therefore, the loss of saliva during most salivary affections can result in different degrees of dehydration in animals, making fluid therapy resuscitation necessary to avoid hypovolemic shock [15].

The most important complication during salivary affections is causing dehydration in animals, so it is necessary to resuscitate them to avoid hypovolemic shock.

The most commonly recorded disorders in buffaloes were parotid salivary gland fistulae, ranula, and cervical sialocele, unlike parotid duct ectasia [3, 6], which has been rarely recorded in buffaloes [8].

The simple, safe, economical, and quick techniques for surgical interventions to treat salivary affections in buffaloes have been recommended by [3, 16], with a higher incidence in adult buffaloes than in calves.

External trauma to the side of the face and incision of an abscess at the cheek are considered the leading causes of salivary fistula [17–19] in humans and animals. In parotid ductal fistulas, continuous discharge of saliva and spontaneous healing are scarce [20]. Since ruminants produce a considerable quantity of saliva, immediate surgical intervention of the salivary fistula is necessary to avoid dehydration and possible infection [17].

Parotid duct fistula can be corrected by duct ligation, destruction of glandular tissue, resection of the gland, repairing the duct, or marsupialization [6, 21]. Ligating the duct proximal to the fistula creates a retrograde pressure buildup within the duct, ultimately ceasing saliva production [22]. Complete duct removal is technically challenging, as the neuromuscular bundles are close to the parotid salivary gland [23]. Partial reconstruction of the parotid duct using a 10 cm segment of polyethylene tube has been recommended in buffaloes and dogs [6, 24].

The ranula can be unilateral [7] or bilateral [12]. The creation of a wide intraoral fistula after marsupialization of ranula/mucocele cases has been reported to be curative without recurrence. Additionally, incisional drainage followed by infusion of irritants into the cystic cavity has been found to destroy the lining membrane and prevent recurrence [7]. Conversely, unilateral removal of the mandibular salivary gland has been indicated on the suspected affected side [8]. Sialoadenectomy of the mandibular salivary gland requires more experience to avoid injuries of the critical structures running along surgical sites, and in most cases, marsupialization provides acceptable results [25]. Although bilateral ranula treatment is simple, it must be differentiated from actinomycosis, foreign body abscess, bottle jaw, foot and mouth disease, and hemorrhagic septicemia [26].

For cervical mucoceles, induced fistulae have been created at the lowest part of the mucocele to facilitate thorough drainage into the oral cavity [14, 27, 28]. Due to the continuous flow of saliva, a longer incision (2 cm, vertical) creates wider, permanent fistulae that can effectively discharge saliva into the oral cavity [6]. Cervical sialoceles tend to recur even after repeated aspiration (removing fluid with a needle); hence, drainage is thought to resolve the problem temporarily [19].

Sialadenectomy (mandibular salivary gland excision) is the best treatment method in dogs and buffaloes [16, 17, 28, 29]. In this study, sialoadenectomy was performed in 30% of the cases that reoccurred after repeated aspiration.

Sialectasis is an uncommon condition characterized by abnormal dilation of the salivary ducts. It can result from various causes, such as several causes, including sialoliths, recurrent infection, trauma, or congenital abnormalities [8, 9, 13]. With sialectasis, a unilateral variable-size swelling is observed on the lateral aspect of the cheek connected to the parotid salivary gland on the caudal side [17, 30, 31].

## Conclusion

To summarize, the most common salivary conditions observed in buffaloes were fistulae, mucoceles, sialoceles, and ectasia, and these were more prevalent among adult buffaloes. Ranulae was the most frequent condition observed in adult buffaloes, closely followed by parotid duct fistulae and cervical sialoceles. Stenson’s duct ectasia, a congenital salivary disease, was the least commonly observed among calves. Surgical intervention is recommended for all salivary affections in buffaloes, resulting in a better prognosis.

## Methods

### Animals

The present study was conducted between January 2011 and November 2022. A total of 135 buffaloes suffering from different surgical affections of salivary ducts were included in the study, as presented in (Table 1). The cases were examined at a private farm station in other localities of Dakahlia Province (82 cases) and those admitted to the Veterinary Hospital of the Faculty of Veterinary Medicine, Mansoura University, Egypt (53 cases). Informed consent was obtained from the owners to participate their animals in the study. The designed protocol for this experiment was accepted by the Mansoura University Animal Care and Use Committee (MU-ACUC) with the approval code MU-ACUC (VM.R.22.11.35).

were contacted by phone, seeking their consent to participate in the study.

All cases were clinically examined before any invasive interventions. Careful case histories, oral examinations, inspections, palpations, aseptic exploratory punctures, and descriptions of clinical signs appearing on the buffaloes were all included in the clinical studies. The aspirated fluid’s consistency, color, and odor were recorded and analyzed in the laboratory. A rehydrated fluid therapy [32] including Lactated Ringer’s solution (500 mL, NASR, Egypt) and normal saline (500 mL, sodium chloride 0.9%, MEPCO, Egypt) were infused throughout the surgical intervention (10–15 mL/kg/hour) in all cases.

The surgical correction was done under the effect of an intramuscular (IM) injection of xylazine HCl (0.05 mg/kg, Xylaject 2%, ADWIA, Cairo, Egypt) followed by local infiltration of lidocaine HCl (Debocaine 2%, El-Nasr Co., Cairo, Egypt) or lidocaine spray (Xylocaine 10%, El-Nasr Co., Cairo, Egypt). The buffaloes were then restrained in lateral recumbency. The mouths of the buffaloes were opened using a mouth gag, and the tongue was pulled out on the lateral side in case of potorid duct ectasia and ranula. Postoperatively, the owners were advised to dress the incision site three times daily with Betadine routinely. Buffaloes were prescribed a daily intramuscular injection of flunixin meglumine (Moraflam, RAA Pharma, Cairo, Egypt) at a dose of 2.2 mg/kg for five days, as well as procaine penicillin (8 mg/kg) and dihydrostreptomycin (10 mg/kg) (Pens&Strep; 1 ml/25 kg; Norbrook®, Newry, United Kingdom). The skin stitches were removed twelve days postoperatively.

### Parotid duct fistula

**Fig. 1:**
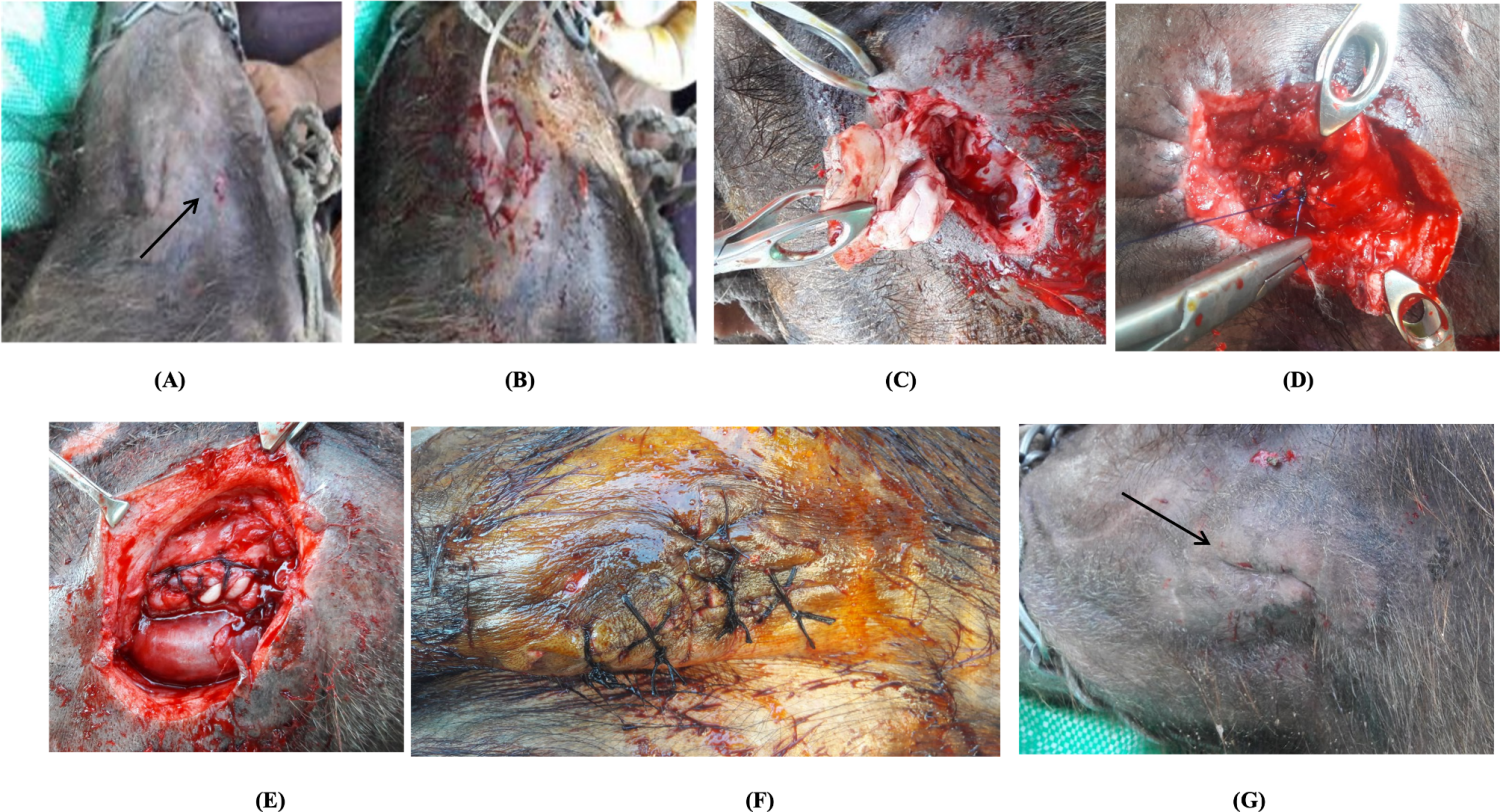
Parotid Salivary Fistula in a Buffalo; **(A)** Narrow opening at the cheek region “black arrow”; **(B)** A baby-sized feeding tube was inserted into the proximal end of the duct followed by an elliptical incision around the fistula; **(C)** Sharp dissection of the subcutaneous tissue and necrotic tissue surrounding the salivary fistula; **(D)** Double ligation; **(E)** blunt dissection of the parotid duct; **(F)** Closure of the wound by suturing of the skin with a cross-suture mattress; and **(G)** Complete wound healing, “black arrow” one month postoperation

Affected buffaloes (n = 44) suffered from the continual discharge of a ropy fluid from the opening at the retroauricular region (12 cases), oral mucosa (eight cases), or skin over the facial cheek (24 cases; Fig. 1A). There was a history of physical trauma in adults (thirty-eight cases) or congenital malformation in calves (six cases, four males and two females).

Fistulectomy was used to treat the parotid duct fistula. In Berife, a baby-sized feeding tube was inserted into the proximal end of the duct, leading towards the gland from the small opening from which saliva was draining (Fig. 1B). Twenty milliliters of methylene blue ink were infused through this tube. An elliptical incision of one centimeter in diameter was made around the fistulous opening, which included the scar tissue. The skin was held, and the subcutaneous tissue, necrotic tissue, and non-healing salivary tracts were sharply dissected until the fistulous tract containing the dye was visible (Fig. 1C). A ligature was placed around the duct at the point where the baby-sized feeding tube was present, using two layers of a simple continuous suture pattern with Prolene or Polygalactin No. 1 (Fig. 1D). Fistulectomy was then performed after ligating the attachment of the fistulous tract to the Stenson’s duct. The duct was immediately ligated after withdrawing the catheter. Transfixation of the salivary duct with the subcutaneous tissue was done using simple interrupted sutures (Fig. 1E). The skin was sutured with a cross-mattress or simple interrupted sutures using Prolene or Silk No. 2 (Fig. 1F). Follow-up of all cases until complete healing was achieved (Fig. 1G).

### Parotid duct ectasia/ sialectasis

**Fig. 2:**
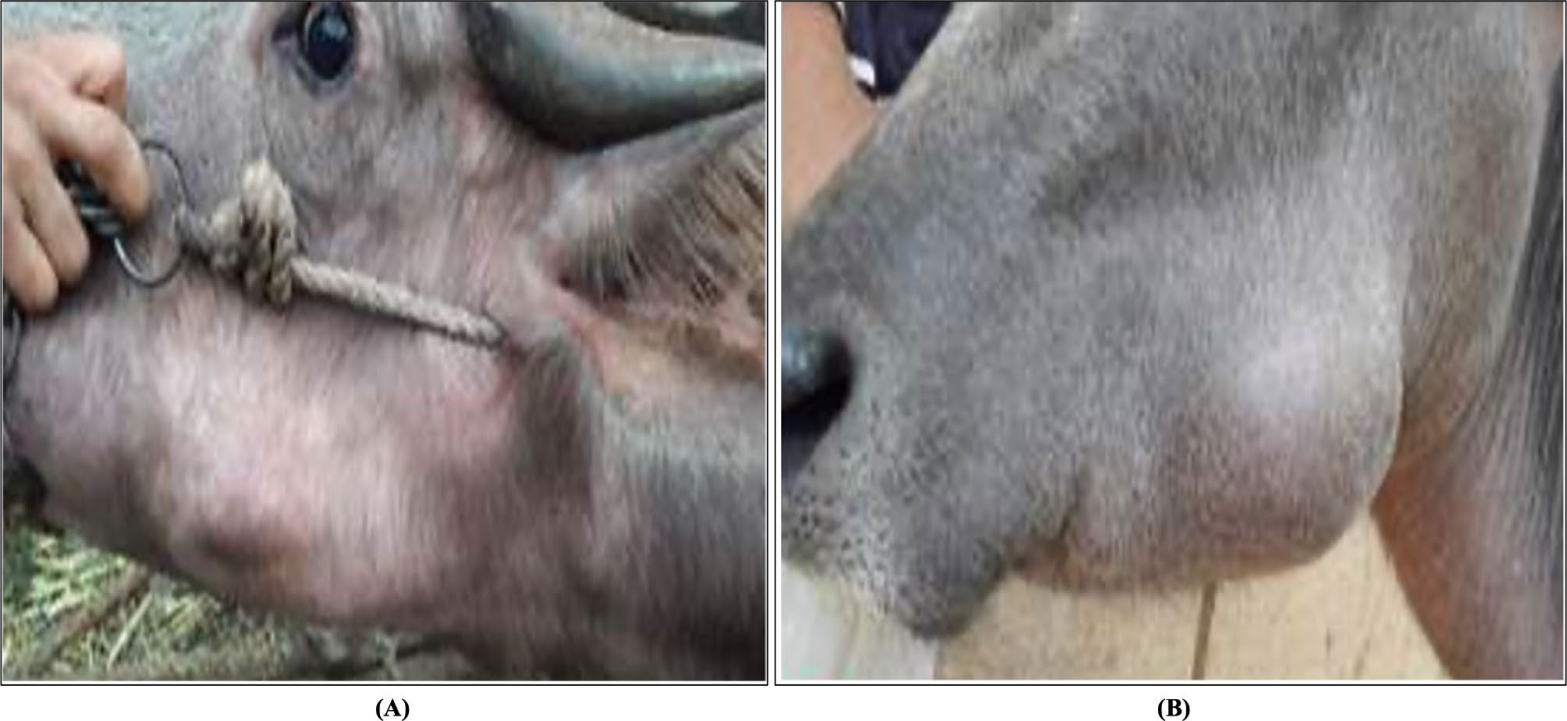
Salivary Duct Ectasia (Sialectasis) in two buffaloes **(A and B)**

Eleven cases appeared as a unilateral, slowly progressive, fluctuating swelling of the parotid region with normal overlying skin appearing. There was no increase in size related to eating, no parotid sialadenitis, no history of facial trauma or allergic disease, and the other major salivary glands were clinically asymptomatic. Furthermore, no lymph node abnormality was observed (Fig. 2A and B). The contained fluid was aseptic centesis for laboratory diagnosis.

Under aseptic conditions, an intra-oral fistula and marsupialization of Stenson’s duct into the mouth cavity were done [6]. An assistant manually pushed the mass from the outside. At the same time, the surgeon made a 2 cm vertical blunt incision on the buccal mucosa using a lancet scalpel No. 22. The wound edges of the vertical surgical incision were sutured to the floor of the buffalo’s mouth on either side using cross-matters suture or simple interrupted suture patterns with a polyglactin 910 (Vicryl; Ethicon; NJ, USA). Retrograde lavage of the cavity was then performed using a diluted povidone-iodine solution (5%). The owners were advised to compress the cheek’s skin thrice daily for five days following the marsupialization.

### Ranula/ mucocele

**Fig. 3:**
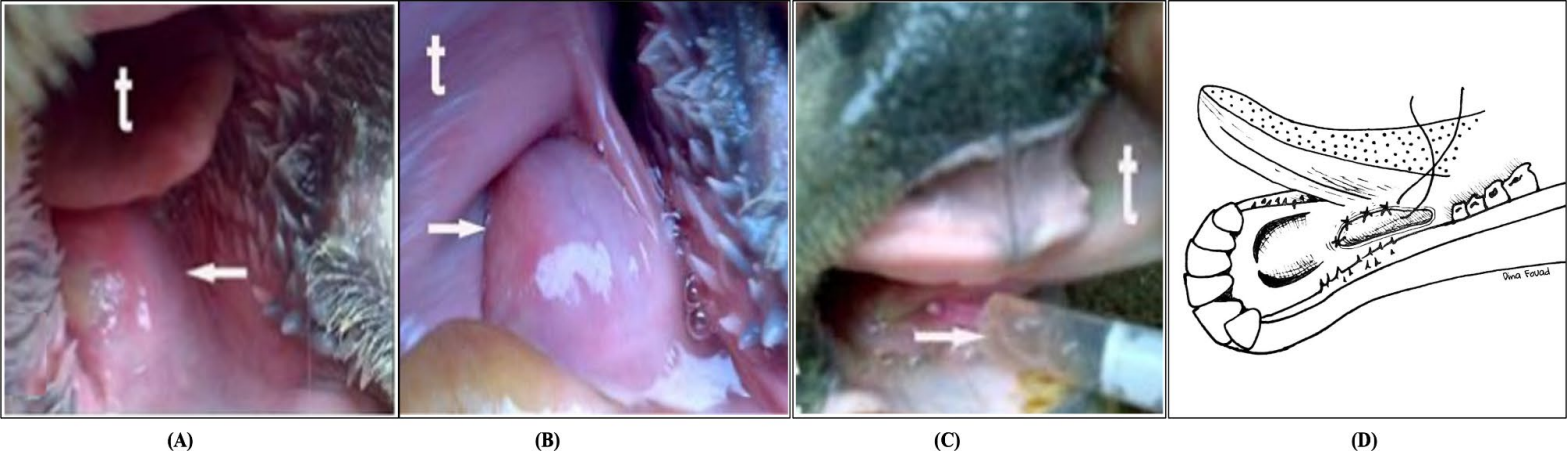
Sublingual Mucocele in a Buffalo. **(A and B arrows)** the swelling under the tongue; **(C arrow)** Needle aspiration of honey-like fluid, t = tongue; and **(D)** Diagram showing sublingual salivary mucocele, which was marsupialized, and the incision wound edges were sutured. (modified from [33])

Ranula or mucocele was recorded as a fluid retention swelling at the frenulum linguae region beneath the tongue (Fig. 3A and B); two cases (4.35%) were registered as bilateral ranula, while the rest were unilateral (95.65%). Acute cases showed decreased appetite with poor prehension of the food, while chronic cases exhibited inappetence and dehydration. The fluid inside the swelling was aspirated under aseptic conditions using a 21-gauge fine needle for analysis (Fig. 3C).

An intra-oral antiseptic wash with 0.05% povidone-iodine was then carried out. An elliptical incision was made in the swelling beneath the tongue, and the internal mucosa of the cyst was curetted to destroy its epithelial covering. Both edges of the cavity were sutured with the tongue mucosa on either side using a 2 − 0 monofilament polyglyconate synthetic absorbable suture (Maxon, Syneture, London, UK) to create a permanent fistula (Fig. 3D).

### Cervical sialoceles

**Fig. 4:**
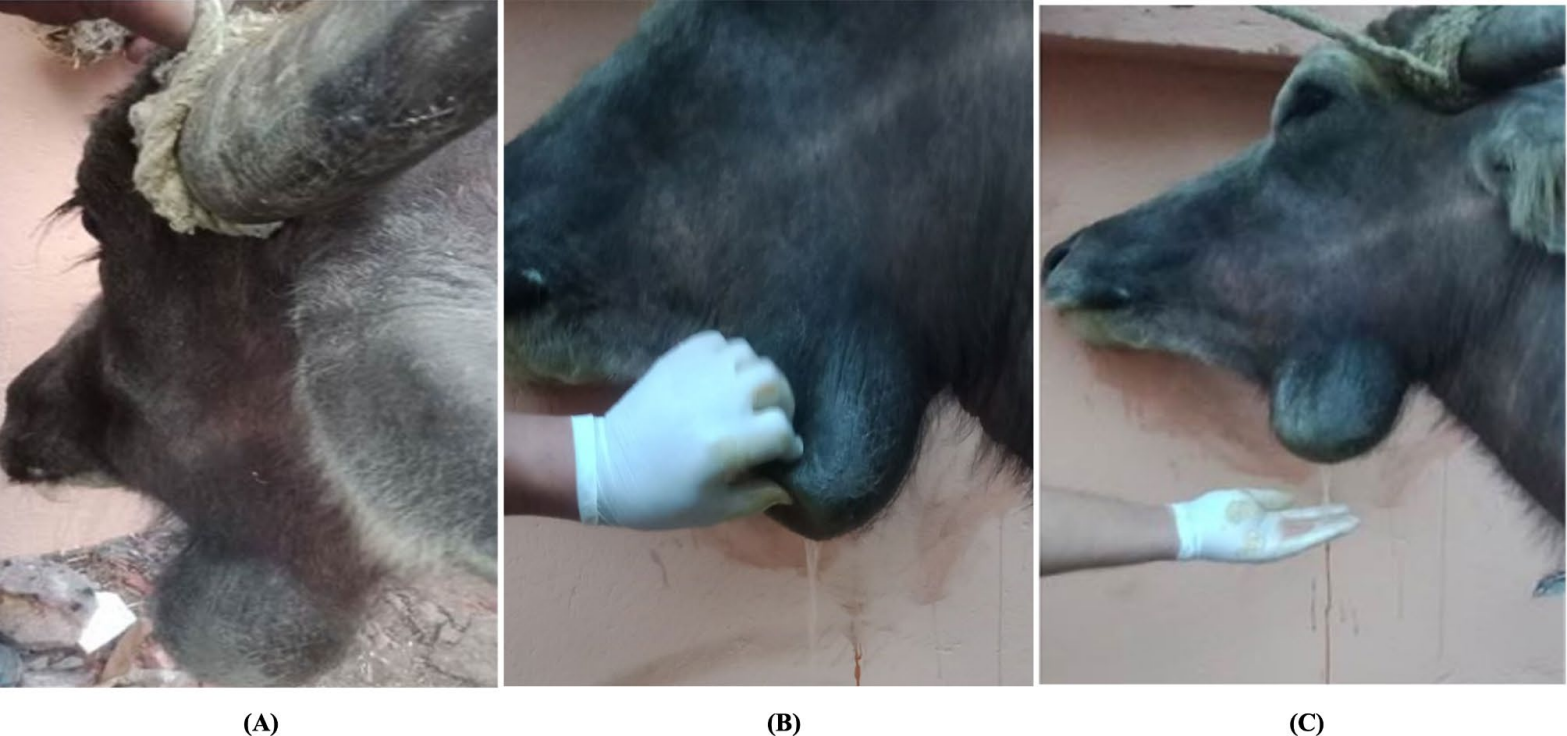
Cervical Sialoceles in a Buffaloe; **(A)** A nodular to frond-like protrusions at the mandibular space; **(B)** Surgical stab incision, and evacuation of content; and (**C)** A clear, viscous, honey-like fluid after stab incision

Cervical sialoceles were diagnosed in 34 buffaloes. The owner had claimed to present with a significant, painless facial, irregular swelling lateral to the mandibular region following a history of trauma. The swelling was soft, fluctuating, and tender to palpation, with nodular to frond-like protrusions (Fig. 4A).

Aseptic preparation of the swelling was done for needle aspiration (18-gauge), followed by a stab incision at the lowest point and evacuation of the viscous fluid contents (Fig. 4B and C). The cavity was then thoroughly flushed with diluted povidone iodine, and after touching it with Betadine, a Penrose drain was applied for ten days.

**Fig. 5:**
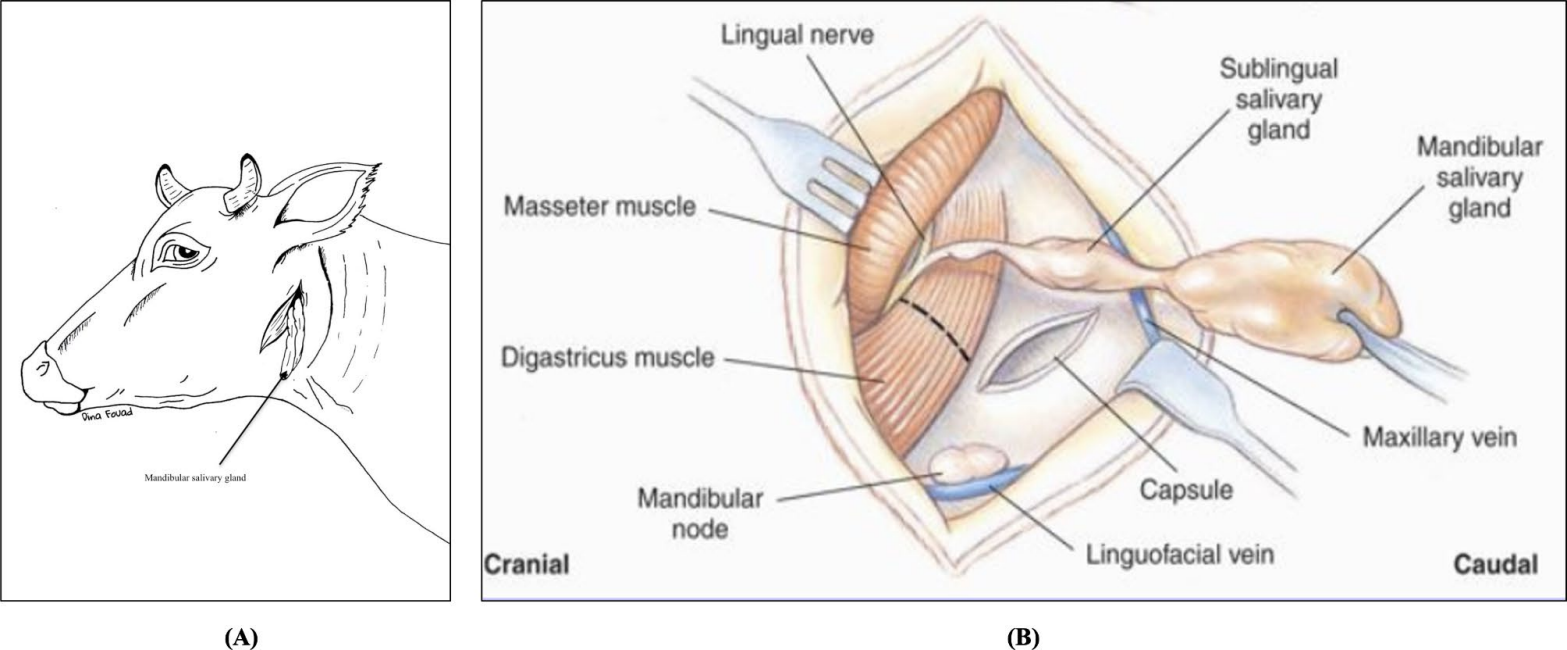
A diagram illustrates the Mandibular Sialadenectomy in Buffaloes; **(A)** The elliptical surgical incision and excision of the lobulated mandibular gland; and **(B)** The anatomical structure of the mandibular and its surrounding structures [33]

The recurrent cases were subjected to sialoadenectomy. After being sedated, the animal was placed in lateral recumbency with the affected side facing upwards. The surgical site was aseptically prepared and infiltrated with local anesthesia. The skin over the salivary gland was elliptically incised, exposing the capsule of the mandibular salivary gland, which was then incised. The mandibular lobulated gland appeared as lobules (ventral, lateral, rostral, and proximal). Lobules of the mandibular gland were carefully grasped, then bluntly and sharply dissected (Fig. 5A and B). The blood vessels supplying the gland and its duct were ligated and severed. The overlying subcutaneous tissues and skin were sutured, as mentioned in [31].

## Data Availability

All data generated or analyzed during this study are included in this article.
